# A chromosome-level genome assembly of the jade perch (*Scortum barcoo*)

**DOI:** 10.1038/s41597-022-01523-y

**Published:** 2022-07-15

**Authors:** Yishan Lu, Ruihan Li, Liqun Xia, Jun Cheng, Hongli Xia, Qiuyao Zhan, Dapeng Yu, Xinxin You, Ruobo Gu, Junmin Xu, Qiong Shi, Chao Bian

**Affiliations:** 1 Guangdong Provincial Key Laboratory of Pathogenic Biology and Epidemiology for Aquatic Animals, Fisheries College, Guangdong Ocean University, Zhanjiang, 524088 China; 2 Guangdong Provincial Engineering Research Center for Aquatic Animal Health Assessment, and Shenzhen Public Service Platform for Evaluation of Marine Economic Animal Seedings, Shenzhen Institute, Guangdong Ocean University, Shenzhen, 518120 China; 3 Shenzhen Key Lab of Marine Genomics, Guangdong Provincial Key Lab of Molecular Breeding in Marine Economic Animals, BGI Academy of Marine Sciences, BGI Marine, BGI, Shenzhen, 518081 China; 4 BGI Education Center, College of Life Sciences, University of Chinese Academy of Sciences, Shenzhen, 518083 China

**Keywords:** Bioinformatics, Genome evolution, Agricultural genetics

## Abstract

Endemic to Australia, jade perch (*Scortum barcoo*) is a highly profitable freshwater bass species. It has extraordinarily high levels of omega-3 polyunsaturated fatty acids (PUFAs), which detailed genes involved in are largely unclear. Meanwhile, there were four chromosome-level bass species have been previous sequenced, while the bass ancestor genome karyotypes have not been estimated. Therefore, we sequenced, assembled and annotated a genome of jade perch to characterize the detailed genes for biosynthesis of omega-3 PUFAs and to deduce the bass ancestor genome karyotypes. We constructed a chromosome-level genome assembly with 24 pairs of chromosomes, 657.7 Mb in total length, and the contig and the scaffold N50 of 4.8 Mb and 28.6 Mb respectively. We also identified repetitive elements (accounting for 19.7% of the genome assembly) and predicted 26,905 protein-coding genes. Meanwhile, we performed genome-wide localization and characterization of several important genes encoding some key enzymes in the biosynthesis pathway of PUFAs. These genes may contribute to the high concentration of omega-3 in jade perch. Moreover, we conducted a series of comparative genomic analyses among four representative bass species at a chromosome level, resulting in a series of sequences of a deductive bass ancestor genome.

## Abstract (summary)

**Table:** 

Measurement(s)	whole genome sequencing
Technology Type(s)	Illumina Sequencing • Oxford Nanopore Sequencing

## Background & Summary

Jade perch (*Scortum barcoo*, NCBI Taxonomy ID: 214431; Fig. 1), also called Barcoo grunter, is a species in the order Perciformes and family Theraponidae. It is native to the Barcoo River of the Lake Eyre Basin, Queensland, Australia^1^. It was introduced into China since 2001. At present, it has become one of the most economically important aquaculture species in several countries including China and Australia due to its rapid growth, highly efficient feed conversion and strong disease resistance^2^. Its flesh is sweet, succulent and slightly flaky without intermuscular bones^3^.

**Fig. 1:**
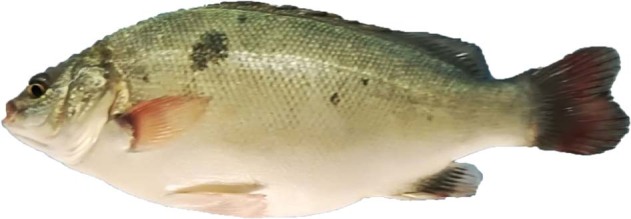
A photo of the collected jade perch (The picture was provided by Hu’s lab^3^).

Meanwhile, jade perch contains highly unsaturated fatty acids. A previous report from the Australian Common Wealth Scientific and Industrial Research Organization (CSIRO) in 1998 revealed that the jade perch contains the highest concentration of omega-3 among 200 examined sea food species^4^. Its omega-3 level was approximately three times of that in Atlantic salmon^4^. Due to these highly nutritional and economical values, jade perch has been widely cultured in many Asian countries, especially in China.

Omega-3 polyunsaturated fatty acids (PUFAs) have a double bond three atoms away from the terminal methyl group. Two representative types of omega-3 PUFAs, eicosapentaenoic acid (EPA) and docosahexaenoic acid (DHA), are both essential fatty acids for the body growth and development of human beings^5,6^. However, humans cannot synthesize these omega-3 PUFAs. These fatty acids can only be obtained from foods such as fish oils. Most fishes have variable abilities to biosynthesize various omgea-3 PUFAs by using fatty acid precursors, such as α-linolenic acids (ALA, 18 carbons with 3 double bonds). This biosynthesis process is mainly controlled by two key enzymes, fatty acy1 desaturases (encoded by *fads*) and elongase of very long-chain fatty acid (encoded by *elovl*)^7–9^.

Mammals have a series of *fads1*, *fads2* and *fads3*, while most of teleosts have only one *fads2*^10,11^. The *fads2* encodes an enzyme to desaturate fatty acids through a double bond among the defined carbons of the fatty acyl chain. There are several types of *fads2* with different desaturated positions, including Δ4 desaturase, Δ5 desaturase, and Δ6 desaturase in various teleosts^12–14^. In addition, the function of Δ8 desaturase of *fads2* was also reported in some studies^15–17^. Moreover, a total of seven members of the *elovl* gene family (*elovl1-7*) have been reported in mammals^18^. Among them, *elovl1*, *elovl3*, *elovl6* and *elovl7* encode enzymes to catalyze the elongation of saturated and mono-unsaturated fatty acids, while *elovl2*, *elolv4* and *elovl5* encode proteins for elongation of PUFAs^19–21^. The functions of fish *elovl2*, *elovl4* and *elovl5* are consistent with those mammalian counterparts^9,22–26^. Recently, two *elovl8* isotypes (*elovl8a* and *elovl8b*; not found in mammals) were first reported in rabbitfish (*Siganus canaliculatus*), and the *elovl8b* has been shown to play an important role in elongation of PUFAs^27^. Due to the high omega-3 PUFA content, the jade perch can be used as a good model for detailed exploration of the biosynthesis pathway of the omega-3 PUFAs.

Furthermore, reconstruction of the ancestral teleost karyotype and models of teleost genome evolution have been proposed previously based on teleost genomes of such as Tetraodon and medaka^28–30^. Medaka genome has been reported to have well conserved teleost ancestor karyotypes without major interchromosomal rearrangements^30^_._ However, even if genomes of four bass species have been sequenced at a chromosome level, the bass ancestral karyotypes were still not deduced.

Here, we applied an integrated strategy of Illumina, Nanopore and Hi-C (high-through-put chromosome conformation capture) sequencing technologies to yield high-quality and sufficient data for subsequent genome assembly, annotation and chromosome construction. Furthermore, we not only localized several key functional genes related to omega-3 PUFA biosynthesis onto chromosomes, but also predicted some potential mechanisms for the high omega-3 PUFA content in the jade perch flesh. We also established the phylogenetic position of jade perch with seven representative teleosts based on single-copy genes, and deduced the bass ancestral karyotypes with a common chromosome translocation in four examined bass species. This jade perch genome assembly, as an important genetic resource, will support in-depth bass biological studies and practical molecular breeding of this economically important fish.

## Methods

### Sample collection

A three-year-old jade perch individual (Fig. 1), bred in a local fishery farm in Foshan City, Guangdong Province, China, was sampled for this study. We collected muscle, spleen, head kidney, body kidney and thymus of this individual for genome and transcriptome sequencing. All samples were freshly frozen in liquid nitrogen, and then stored at –80 °C until use. This sampling pipeline was approved by the Institutional Review Board on Bioethics and Biosafety of BGI-Shenzhen, China (No. FT18134).

### DNA extraction and genome sequencing

Genomic DNA was extracted from the muscle tissue by using a Nucleic Acid Kit (Qiagen, Germantown, MD, USA) following the manufacturer’s instructions. These DNA samples were used to construct libraries for subsequent sequencing on Illumina (Illumina Inc., San Diego, CA, USA) and Nanopore (Oxford Nanopore Technologies, Oxford, UK) platforms. In brief, three paired-end libraries with different insert sizes (270 bp, 500 bp and 800 bp) were constructed and then sequenced on an Illumina HiSeq X Ten platform. About 118.3 Gb of raw reads with paired-end 150 bp were generated. Approximately 109.9 Gb of clean reads were retained for further assembly after removal of low-quality or duplicated reads and adapter sequences by SOAPfilter v2.2^31^ (parameters: -z -p -g 1 -M 2 -f 0). For Nanopore library, the gDNAs were size-selected (10–50 kb) with a Blue Pippin system (Sage Science, Beverly, MA, USA) and processed using the Ligation Sequencing 1D kit (SQKLSK109, Oxford Nanopore Technologies) according to the manufacturer’s instructions. About 82.6 Gb of raw data with fast5 format were collected. After removal of low-quality reads (mean_qscore < 7), 40.0-Gb reads with a mean length of 21.2 kb passed the quality control. Correction of Nanopore reads was then performed by mapping the Illumina clean reads to the Nanopore sequence data using the LoRDEC^32^ program with default parameters.

### RNA extraction and transcriptome sequencing

Muscle, spleen, head kidney, body kidney and thymus tissues of the jade perch were homogenized in the TRIZOL Reagent (Invitrogen, Carlsbad, CA, USA). Library construction and transcriptome sequencing were performed on an Illumina HiSeq X Ten platform in accordance with the manufacturer’s protocols. A total of 20.0 Gb data (about 4.0 Gb for each tissue) were generated for transcript and genome annotation.

### Estimation of the genome size

We estimated the genome size of jade perch by using the routine 17-mer frequency distribution analysis^33,34^. Cleaned Illumina sequences with the insert size of 800 bp were used as the input file. The genome size was calculated according to the following equation: genome size = k-mer number/the estimated k-mer depth. In the case of sufficient data, the k-mer frequency distribution follows a normal Poisson distribution. The peak of the practical k-mer distribution curve is considered as the expected k-mer depth. The estimated genome size of jade perch is about 692.7 Mb, with 5.8 × 10^10^ k-mers and a peak k-mer depth of 84.

### *De novo* genome assembly and chromosome construction

For the *de novo* genome assembly, we adopted a hybrid strategy of combining both the clean Illumina and Nanopore reads. Firstly, we employed Platanus v1.2.4^35^ (parameters: -k 29 -d 0.3 -t 16 -m 450) to generate initial contigs based on the clean Illumina reads. Subsequently, these contigs and error-corrected Nanopore long reads were both used as input files for the DBG2OLC^36^ assembly pipeline (parameters: LD 1 k 17 KmerCovTh 6 MinOverlap 60 AdaptiveTh 0.012). Finally, a high-quality genome of the jade perch was assembled. The scaffold-level genome of 657.7 Mb was firstly assembled, accounting for 94.9% of the estimated genome size. We also evaluated the completeness of this assembly by using the BUSCO v5.2.2 assessment^37^. The BUSCO evaluation calculated the genome module benchmark value to be C: 97.9%, including S: 95.6%, D: 2.3%, F: 0.1%, M: 2.0%, and n = 3,640 (C: complete, S: single-copy, D: duplicated, F: fragmental, M: missed, and n: total BUSCO groups of Actinopterygii_ odb9 data set). A total of 3,565 out of the 3,640 (97.9%) from Actinopterygii gene set had been identified completely in the assembled genome of jade perch (Table 1).

**Table 1:** Statistics of the assembled genome for the Jade perch.

Genome assembly statistics	Data	
Total length	657,708,326	
Largest contig length	13,630,233	
N50 length (contig)	4,771,844	
N50 length (scaffold)	28,580,765	
Total ( > 2 kb)	501	
GC rate (% of genome)	0.4	
repeat elements (% of genome)	19.7	
**BUSCO genome completeness score**	**Data**	**Ratio**
Complete and single-copy BUSCOs (C)	3,481	95.6%
Complete and duplicated BUSCOs	84	2.3%
Fragmented BUSCOs (F)	5	0.1%
Missing BUSCOs (M)	70	2.0%
Total number of Actinopterygii orthologs	3,640	

**Fig. 2:**
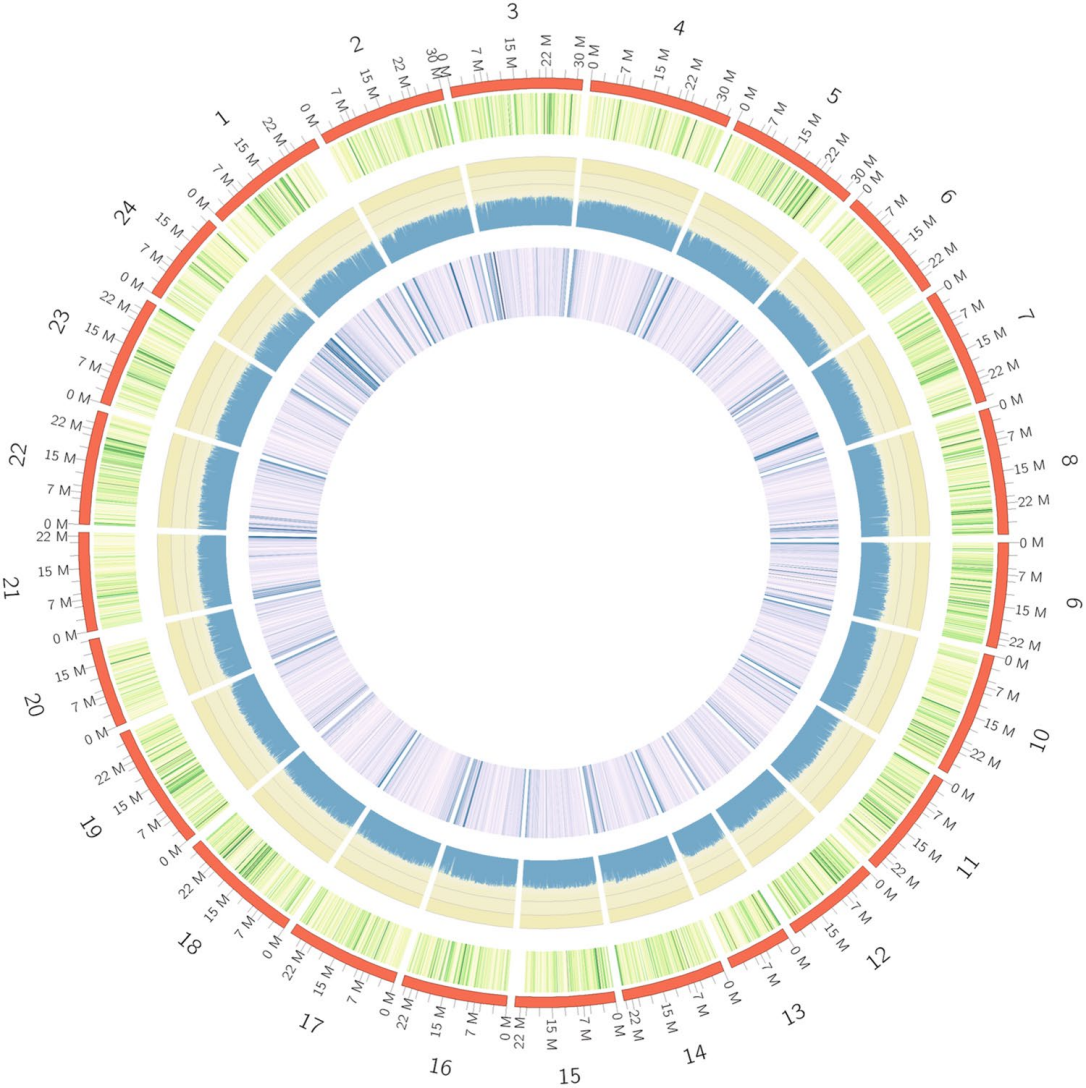
Characterization of the assembled genome of jade perch. From the inner to the outer layers: repeat element abundance (violet), GC rate (blue), gene abundance (green), and chromosomes with scale (red).

The Hi-C technique was applied to construct a chromosome-level genome of jade perch. In brief, muscle sample was fixed by formaldehyde. The restriction enzyme (*Mbo I*) was used for digestion of extracted genomic DNAs, followed by repairing of the 5′ overhangs with a biotinylated residue. A paired-end library with an insert size of approximately 300 bp was constructed. About 48.0 Gb of Hi-C reads were generated by sequencing on the HiSeq X ten platform. These Hi-C reads were mapped onto the assembled scaffolds by using Bowtie2^38^ (parameters: --very-sensitive -L 30 --score-min L,-0.6,-0.2 --end-to-end –reorder). We then obtained all valid pair information with scaffold linkage from the results of HiC-Pro v2.8.0^39^ with default parameters. Juicer v1.5^40^ (parameter: chr_num 24) and 3d-DNA v170123^41^ (parameters: -m haploid -r 2) were applied to anchor these scaffolds into chromosomes. We also utilized Juicebox v1.11.08^42^ to fix error-joins and removed duplicated contigs. The 3D-DNA pipeline employed the correction file from Juicebox to generate the final chromosome-level genome assembly. By using these Hi-C data, we constructed 24 chromosomes with a total length of 642.9 Mb, accounting for 97.7% of the scaffold-level genome (Fig. 2). The Contig and scaffold N50 values of this final chromosome-level genome assembly are 4.5 Mb and 28.6 Mb, respectively.

### Repeat annotation

We employed two approaches, including *ab initio* prediction and homology annotation, to detect repetitive elements in the assembled genome of jade perch. For the *ab initio* prediction, we applied RepeatModeler v1.0.8^43^ and LTR-FINDER v1.0.6^44^ with default parameters to detect various types of repetitive elements. Subsequently, RepeatMasker v4.0.6^45^ with default parameters was employed to construct a new library based on the Repbase TE v21.01^46^. Additionally, Tandem Repeats Finder^47^ (parameters: 2 7 7 80 10 50 2000 -d -h) was used to identify tandem elements. For the homology prediction, RepeatMasker v4.0.6^45^ and RepeatProteinMask v4.0.6^45^ with default parameters were employed to search repeat elements in the genome assembly based on the repeat library from the *ab initio* prediction. We finally predicted about 129.5 Mb of repetitive sequences, accounting for 19.7% of the assembled jade perch genome. This ratio is at the middle level between European seabass (21.5%)^48^ and large yellow croaker (18.1%)^49^, both of which belong to Percomorphaceae.

### Gene prediction and functional assignment

To obtain a fully annotated gene set, we combined three methods to predict protein-coding genes, including *de novo* prediction, homology-based annotation and transcriptome-based annotation. For the *de novo* prediction, Augustus v2.5^50^ with default parameters was used to predict coding regions on the repeat-masked assembly with default parameters. For the homology-based prediction, protein sequences of seven representative teleosts, including zebrafish (*Danio rerio*), threespine stickleback (*Gasterosteus aculeatus*), medaka (*Oryzias latipes*), Japanese pufferfish (*Takifugu rubripes*), green-spotted pufferfish (*Tetraodon nigroviridis*), Southern platyfish (*Xiphophorus maculatus*), and European seabass (*Dicentrarchus labrax*), were downloaded from the public NCBI database (release 75) for mapping onto the jade perch genome assembly by TBLASTn^51^ with an e-value ≤ 10^-5^. Subsequently, GeneWise v2.2.0^52^ (parameters: --blast_eval 1e-5 --align_rate 0.5 --extend_len 500) was applied to identify gene structures on the basis of the tBLASTn alignments. For the transcriptome-based annotation, pooled RNA-seq reads were mapped onto the jade perch genome by using Tophat v2.1.1^53^. Cufflinks v2.2.1 (http://cole-trapnell-lab.github.io/cufflinks/) was employed to detect gene structures on the RNA-seq alignments. All gene sets from the above-mentioned three approaches were merged by MAKER^54^ (parameters: max_dna_len = 300000, min_contig = 500, pred_flank = 500, AED_threshold = 1, split_hit = 30000, single_exon = 1, single_length = 250, tries = 2) to generate a final non-redundant gene set. It was functionally annotated by aligning against three public databases, including SwissProt^55^, TrEMBL^56^, and KEGG (Kyoto Encyclopedia of Genes and Genomes)^57^. Gene ontology (GO) annotation was performed by the InterProScan program^58^.

We predicted a total of 26,905 protein-coding genes (close to the 26,719 of European seabass; Table 2) with a mean length of 15,168.9 bp. Each gene has an average number of 9.9 exons, with a mean length of 179.6 bp. About 97.9% (26,327 genes) of the total predicted genes were assigned with at least function annotation (Table 3).

**Table 2:** Statistics of the predicted protein-coding genes in the jade perch genome.

Parameter	Species	Gene Number
Homolog	*D. rerio*	25,974
	*D. labrax*	35,157
	*L. calcarifer*	27,802
	*L. maculatus*	30,953
	*M. salmoides*	21,845
	*O. latipes*	26,659
	*G. aculeatus*	26,738
Transcriptome		26,654
MAKER		26,905

**Table 3:** Function annotation of genes by multiple methods.

Parameter	Number	Percentage (%)
Total	26,905	100.0
Swissprot	20,706	77.0
KEGG	24,104	89.6
TrEMBL	26,298	97.7
GO	20,603	76.6
Overall	26,327	97.9

### Ortholog and phylogenetic analyses

Reference protein sequences of eight representative species, including Asian arowana (*Scleropages formosus*), zebrafish, medaka, Asian seabass (*Lates calcarifer*), threespine stickleback, European seabass, American black bass (*Micropterus salmoides*), and Chinese seabass (*Lateolabrax maculatus*), were downloaded from NCBI database (release 75). These protein sets and our jade perch protein set were filtered by removing those protein sequences with less than 50 amino acids. All to all blast was performed by BLASTP with an e-value ≤ 10^-5^ to identify homologous sequences^51^. These protein sets were then clustered into gene families by using OrthoMCL (v2.0.9)^59^ with default parameters.

To identify the phylogenetic position of jade perch, we employed MUSCLE v3.8.31^60^ to align single-copy orthologous genes from the used nine species. Subsequently, these protein sequences were converted to their according coding sequences. Alignments were concatenated to form a single supergene for each species. The alignments of supergenes were then used to construct a Maximum Likelihood (ML) tree by PhyML v2.4.4^61^ (parameters: -rates gamma -a e -c 4 -t e). MCMCtree^62^ (parameters: -model HKY85, -clock independent rates, -seed -1) in the PAML package v4.9 was employed to calculate the divergence times among the selected nine fish species.

**Fig. 3:**
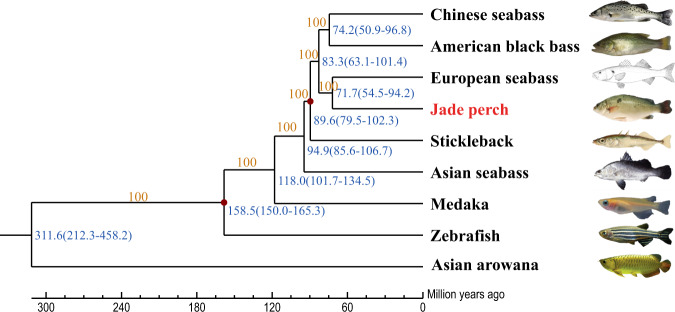
Divergence time tree of nine representative teleosts. Asian arowana was used as the outgroup. The pictures of Asian seabass, European seabass and Chinese seabass were cited from Randall (1997, Wikipedia Commons), Bauchot (1987, Wikipedia Commons) and Chen’s study^73^, respectively. Orange numbers represent the bootstrap values. Blue numbers represent the divergence times. Red dots represent those confirmed divergence times from the TimeTree (http://www.timetree.org/).

A phylogenetic tree was constructed (Fig. 3), and it demonstrated that jade perch and European seabass were clustered into one clade with a bootstrap value of 100. The close relationship between these two bass species is consistent with their traditional morphological classification. Their divergence time was estimated to be 71.7 million years ago (see Fig. 3).

### Reconstruction of ancestral chromosomes from four representative bass species

**Fig. 4:**
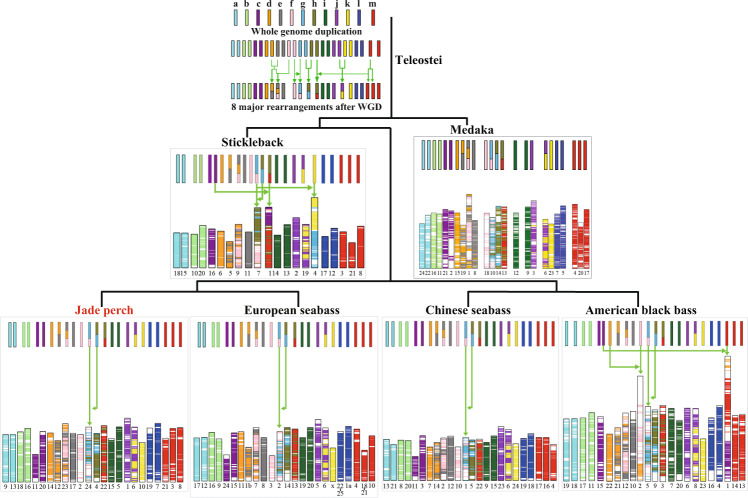
Evolution of six representative fish chromosome karyotypes. Thirteen ancestral chromosomes are marked with various color bars. Genomic regions from the same ancestral chromosomes were presented in the same color. Green arrows represent translocation, fusion and fission events.

The ancestral teleost karyotype was estimated to contain 13 pairs of potential chromosomes that were marked as Ancestor Chromosomes a~m in previous studies^29,63^. Each protein set of four representative bass species, including jade perch, Chinese seabass, American black bass and European seabass^64^, and stickleback was aligned to that of the predicted ancestor chromosomes respectively by using BLASTP (e-value < 1 × 10^−10^). We then identified the reciprocal best-hit alignments between each of the four bass species and the ancestor chromosomes. Finally, chromosome fissions, fusions and translocations were identified and demonstrated by using SVG in Perl (Fig. 4). We determined that jade perch, European seabass, American black bass and Chinese seabass have well conserved most of the ancestral chromosome karyotype (Fig. 4). A common chromosome translocation appeared in their chromosomes, when compared to the ancestral chromosomes. As to our jade perch genome assembly has more complete sequences than the reported genomes of other bass species (Table 4), it could be the best reference genome assembly for studying the Perciformes evolution.

**Table 4:** Statistics of the genome assemblies for representative bass species.

Latin name	Common name	Contig N50 (Mb)	Scaffold N50 (Mb)	Genome size (Mb)	Chr number	Anchored pseudo-chromosomes (%)	Complete BUSCOs (%)	Gene number
*Micropterus salmoides*	American black bass	1.2	36.5	964.0	23	89.4	97.2	23,701
*Dicentrarchus labrax*	European seabass	0.05	5.1	675.0	24	86.0	96.0	26,719
*Lates calcarifer*	Asian seabass	1.0	25.0	668.4	24	87.8	96.8	22,184
*Lateolabrax maculatus*	Chinese seabass	0.03	28.6	668.0	24	77.7	97.0	22,015
*Scortum barcoo*	Jade perch	4.7	42.5	657.7	24	97.7	97.9	26,905

### Identification of omega-3 PUFA biosynthesis related genes

Two key gene families (*fads* and *elovl*) related to biosynthesis of omega-3 PUFAs were identified in the jade perch. Zebrafish *fad* and *elovl* protein sequences (Genbank accession numbers: Fads2, NC_007136.7; Elovl1a, NC_007113.7; Elovl1b, NC_007122.7; Elovl2, NC_007135.7; Elovl4a, NC_007127.7; Elovl4b, NC_007134.7; Elovl5, NC_007124.7; Elovl6, NC_007125.7; Elovl7a, NC_007119.7) were used as the queries to align against the jade perch genome by using TBLASTn^51^. GeneWise v2.2.0^52^ (parameters: --blast_eval 1e-5 --align_rate 0.5 --extend_len 500) was used to predict gene structures based on these alignments.

**Fig. 5:**
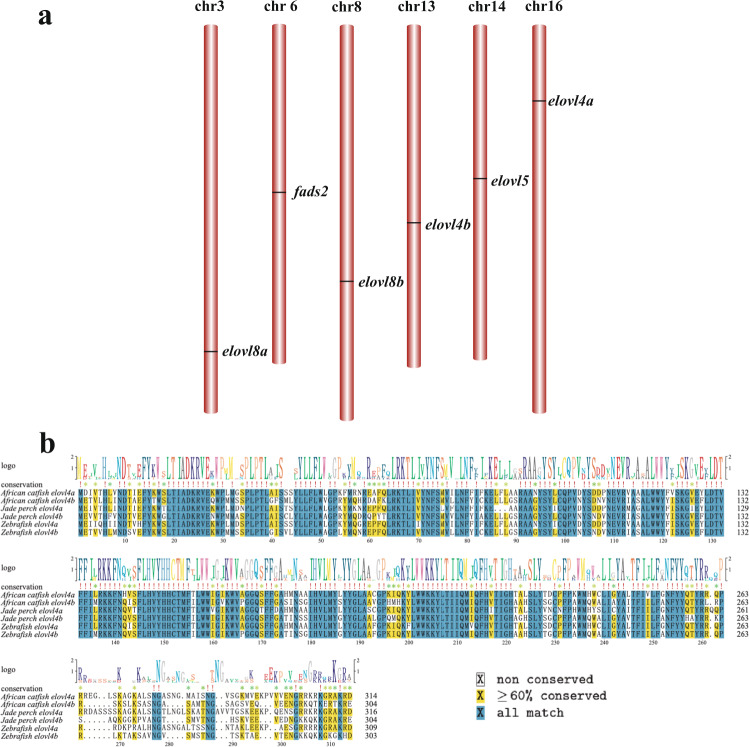
Characterization of the core genes for biosynthesis of polyunsaturated fatty acids. (**a**) Critical *fads* and *evovl* genes were localized in the jade perch genome. (**b**) Elov4a and Elov4b protein sequences from jade perch, African catfish and Zebrafish were aligned for comparison.

We identified the core genes for omega-3 PUFA biosynthesis in the jade perch genome (Fig. 5a). As reported in the majority of teleosts^11^, e*lovl2* was absent while one copy of *fads2* and one *elovl5* were present in jade perch (chr 6 and chr 14, respectively). Two *elovl4* genes (*elovl4a* and *elovl4b*) were localized in the chr16 and chr13, respectively (Fig. 5a). This number, however, is different from most of the reported fishes, which usually have only one copy of *elovl4* gene^11,16,25,65–68^. In addition, the *elovl4a* of jade perch shared a higher identity (82.5%) with its ortholog in African catfish (*Clarias gariepinus*; Fig. 5b), than that in zebrafish (79.7%). This implies that jade perch, similar to African catfish^69^, may have an efficient ability to synthesize Omgea-3 PUFAs for their accumulation in the flesh.

Recently, a novel elongase (termed as *elovl8*) was reported in rabbitfish with two isotypes (*elovl8a* and *elovl8b*), and functional experiments revealed that *elovl8b*, but not *elovl8a*, has a capacity to elongate C18 and C20 PUFA precursors to produce a longer PUFA^27^. Similarly, we identified both *elovl8a* and *elovl8b* genes in the jade perch genome (chr 3 and chr 8, respectively; see Fig. 5a). At least one of both elongases may participate in the high PUFA accumulation in jade perch flesh.

Previous studies reported that *fads2*, *elovl2*, *elovl5*, and *elovl8b* were primarily distributed in liver, while *elovl4a*, *elovl4b* and *elovl8a* were mainly present in brain, eye and gonad^9,25,27,66,69,70^. We therefore speculate that these gene families of both *fads* and *elovl* may elevate the biosynthesis content of PUFAs in jade perch, resulting in abundant omega-3 PUFAs in at least its flesh.

Furthermore, these PUFA biosynthesis related genes are individually located on different chromosomes (Fig. 5a). This scattered pattern suggests that these gene copies could not be generated by tandem duplication, instead more possibly by fish-specific genome duplication.

## Data Records

The genome assembly and annotation files were deposited in China National GeneBank (CNGB) under the accession number CNP0002889^71^ and NCBI under the accession number SRP370737^72^. Raw reads of genome and transcriptome generated in the present study were deposited in the NCBI under the accession number SRP370737^72^.

## Technical Validation

The agarose gel electrophoresis was used to check the quality of extracted DNA molecules. The main band is around 20 kb, and the DNA spectrophotometer ratios (260/280) were over 1.8. The quality of purified RNA molecules was examined by Nanodrop ND-1000 spectrophotometer (LabTech, Corinth, MS, USA) as the absorbance > 1.7 at 260 nm/280 nm and 2100 Bioanalyzer (Agilent Technologies, Santa Clara, CA, USA) as the RIN of 8.0. We further evaluated the completeness of the jade perch genome assembly by using BUSCO v5.2.2, and determined that 97.9% of BUSCO genes were complete.

## Data Availability

All commands and pipelines used for the genome and transcriptome analyses were performed according to those manuals and protocols of the applied bioinformatics software.
